# Hands-on training about overfitting

**DOI:** 10.1371/journal.pcbi.1008671

**Published:** 2021-03-04

**Authors:** Janez Demšar, Blaž Zupan

**Affiliations:** 1 Faculty of Computer and Information Science, University of Ljubljana, Ljubljana, Slovenia; 2 Department of Molecular and Human Genetics, Baylor College of Medicine, Houston, Texas, United States of America; SIB Swiss Institute of Bioinformatics, SWITZERLAND

## Abstract

Overfitting is one of the critical problems in developing models by machine learning. With machine learning becoming an essential technology in computational biology, we must include training about overfitting in all courses that introduce this technology to students and practitioners. We here propose a hands-on training for overfitting that is suitable for introductory level courses and can be carried out on its own or embedded within any data science course. We use workflow-based design of machine learning pipelines, experimentation-based teaching, and hands-on approach that focuses on concepts rather than underlying mathematics. We here detail the data analysis workflows we use in training and motivate them from the viewpoint of teaching goals. Our proposed approach relies on Orange, an open-source data science toolbox that combines data visualization and machine learning, and that is tailored for education in machine learning and explorative data analysis.

## Author summary

Every teacher strives for an a-ha moment, a sudden revelation by the student who gained a fundamental insight she will always remember. In the past years, authors of this paper have been tailoring their courses in machine learning to include material that could lead students to such discoveries. We aim to expose machine learning to practitioners–not only computer scientists but also molecular biologists and students of biomedicine, that is, the end-users of bioinformatics’ computational approaches. In this article, we lay out a course that aims to teach about overfitting, one of the key concepts in machine learning that needs to be understood, mastered, and avoided in data science applications. We propose a hands-on approach that uses an open-source workflow-based data science toolbox that combines data visualization and machine learning. In the proposed training about overfitting, we first deceive the students, then expose the problem, and finally challenge them to find the solution. In the paper, we present three lessons in overfitting and associated data analysis workflows and motivate the use of introduced computation methods by relating them to concepts conveyed by instructors.

## Introduction

Machine learning is one of the critical bioinformatics technologies [1]. Applications of machine learning span across the entire spectra of molecular biology research, from genomics, proteomics and gene expression analysis to development of predictive models through large-scale integration [2]. The advantage of machine learning methods is that they can automatically formulate hypotheses based on data, with occasional guidance from the researcher. In its flexibility lies the machine learning’s strength–and its greatest weakness. Machine learning approaches can easily overfit the training data [3], expose relations and interactions that do not generalize to new data, and lead to erroneous conclusions.

Overfitting is perhaps the most serious mistake one can make in machine learning. In his excellent review, Simon *et al*. [4] points out that a substantial number of the most prominent early publications on gene expression analysis overfitted the data when reporting predictive or clustering models. Mistakes of these kinds are today rare, yet the problem with overfitting persists [3,5]. It is thus essential to convey the intricacies and facets of overfitting to students that are taught about data science, and we should include lectures on overfitting already within introductory courses of machine learning.

Our aim here is to show that one can effectively explain overfitting during a short hands-on workshop. Our primary audience are students of molecular biology and biomedicine, not students of computer science or math. Essential to such teaching is a tool that supports the seamless design of data analysis pipelines that encourages experimentation and explorative data analysis. Since 2005, we have been designing such a data science toolbox. Orange (http://orange.biolab.si) [6] is a visual programming environment that combines data visualization and machine learning. In the past years, we have been tailoring Orange towards a tool for education (e.g., [7,8]). We have used it to design short, practical hands-on workshops. To boost motivation and interest, we use problem-based teaching. There, we first expose students to data and problems, rather than to theory and mathematical background of machine learning, a type of training that would be more appropriate to computer science majors.

In the following, we introduce a teaching approach for hands-on training and exploration of overfitting. We lay out pedagogical methods and the training approach first and then continue with a presentation of the short course that includes three different cases of overfitting. We conclude with a discussion about further details on the engagement of tutoring staff and placement of this course within computational biology curricula.

## Approach

The lecture we propose here was designed to comply with a structured pedagogical approach, requires a specific data science platform, and uses hands-on training. We briefly explain each of these items below.

### Didactic methods

When discussing overfitting, we use the following didactic approach.

- Introduce a seemingly reasonable analytic procedure for fitting and testing the model.
- Demonstrate that the procedure gives overly optimistic results, which is most efficiently done by showing that it allows modeling randomized data.
- Analyze why and how this happens.
- Show the correct approach.
- Emphasize the take-home message.

The most crucial step is the third because it leads students to a deeper understanding of the problem and lets them apply similar reasoning to other situations they may encounter.

### Software platform

**Fig 1:**
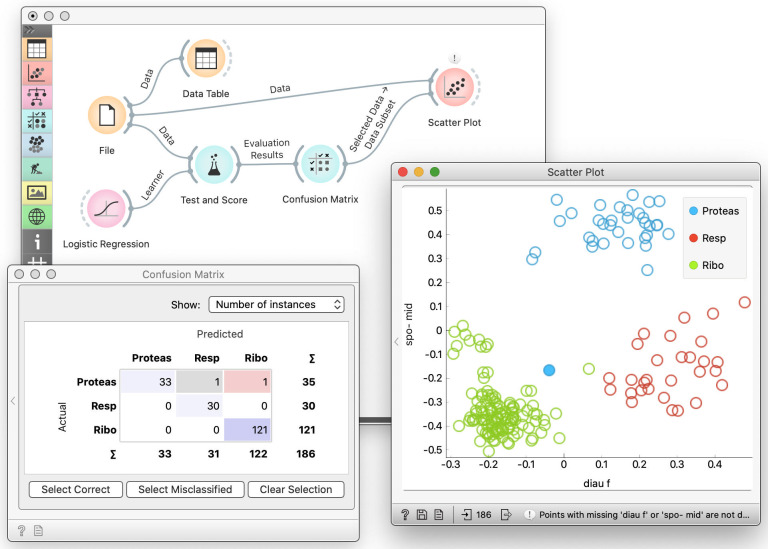
Orange data science toolbox. Orange provides data analysis components, also called widgets, assembled into a data analysis workflows through visual programming. The components typically encapsulate some data processing or modeling methods; they receive the input and submit the results to the output. Widgets in Orange are represented with icons with an input slot on the left and the output slot on the right. Users place widgets on the canvas and connect the inputs and outputs of the widgets. In this way, they define the data and information processing pipeline. The system processes the workflow on-the-fly: as soon as the widget receives the information, it would handle it and send out the results. In the workflow shown on the figure, the data pipeline starts with reading the data (File widget) and passes it to cross-validation (Test and Score), which also receives a learning algorithm on its input. Double-clicking the widget exposes its content. For instance, we pass cross-validation results to the Confusion Matrix, which shows that logistic regression misclassified only two data instances. We use the Scatter Plot to show the entire data set and also display selected data from the Confusion Matrix. Any change in selection in the Confusion Matrix would change its output. This would trigger the change in the Scatter Plot. With this composition of components, we turn this workflow into a visual explorative environment for examining cross-validation results.

For training in machine learning, we use the data science toolbox called Orange (http://orange.biolab.si) [6,8,9]. Orange is an open-source, cross-platform data mining and machine learning suite. It features visual programming as an intuitive means of combining data analysis and interactive visualization methods into powerful workflows (Fig 1). Visual programming enables users who are not programmers to manage, preprocess, explore, and model data. With many functionalities aboard, this software can make data mining and machine learning easier for novice and expert users.

### Teaching approach

The teaching approach we propose is hands-on training. Students follow the lecture by working on their computer or laptop. The lecturer uses the projector and explains the concepts from the lectures by performing data exploration and analysis. There are no slides. For additional explanations, the lecturers are encouraged, where possible, to provide the students with data exploration examples, using the same data science software. For larger classes, the lecturer is accompanied by assistants who help the students who are stuck or have any questions about their workflows.

## Three cases of overfitting

We here present three ways to overfit the model to the data. We assume that at this point students already know at least some machine learning and visualization-related algorithms. In this article, we use classification trees, logistic regression, and t-SNE, though we could substitute them with other alternatives. We also refer to classification accuracy and information gain. If the students are not familiar with these scoring techniques, a brief introduction will suffice during the workshop.

### Testing on training data

Any hypothesis formulated from some data, and that fits that data well, will seem correct when verified on this same data. While this looks obvious, researchers from fields other than artificial intelligence, like biology, often forget that the essence of AI is precisely an automated generation of hypotheses based on data. Furthermore, though students may have heard the mantra never to test the model on the data from which it was derived, they may consider it merely a recommendation whose violation is wrong in principle, but will not have any significant consequences.

### Analytic procedure

For this demonstration, we use the yeast data with 186 genes (data instances) whose expression was observed at 79 different conditions (features). The data also includes a class variable, which for each gene reports on one of the three gene functions. This data set comes from Brown *et al*. [10], the first work that used supervised machine learning for gene function prediction. The data set used in their paper includes more genes, from which we have retained only those from the three most frequent classes (c.f. [11]).

For this example, the number of features in the data set must be comparable to the number of data instances, which should allow simple models to overfit the training data. Here, we choose classification trees because they are sufficiently prone to overfitting.

**Fig 2:**
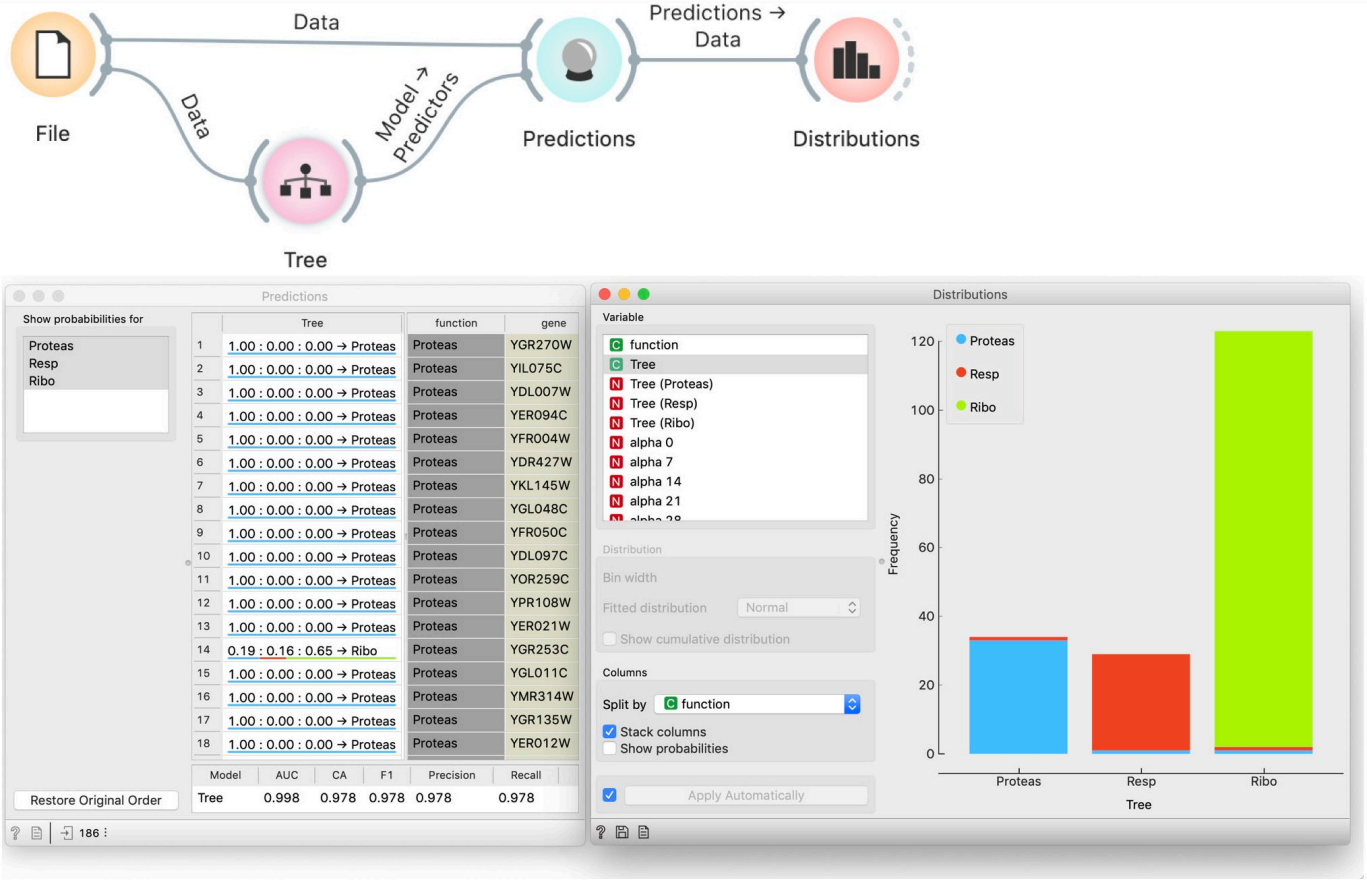
Incorrect evaluation of models. The tree is tested on the data from which it was induced. The Distribution widget shows perfect correspondence between the predicted and actual gene functions.

We load the data and feed it to a tree inducer, which outputs a tree model (Fig 2). The Predictions widget takes the data, uses the tree to predict the target variable, and outputs a data table augmented with a column that stores predictions. We feed this data to Distributions, which we use to visually assess the model’s correctness. We set up the widget’s parameters to show tree predictions and split them by genes’ actual function and stack columns. (Column stacking will become important later.) We visually confirm that most genes indeed belong to the groups into which they are classified. Using such visualization may be better than showing just numbers like classification accuracy because it is more intuitive. Students can, however, still check the classification accuracy and other scores in the Predictions widget. Quantitative information of what we see in Distributions is available by connecting the Confusion Matrix widget to Predictions. However, we do not recommend overwhelming the students with too many concepts at this moment.

### Demonstration of incorrectness

**Fig 3:**
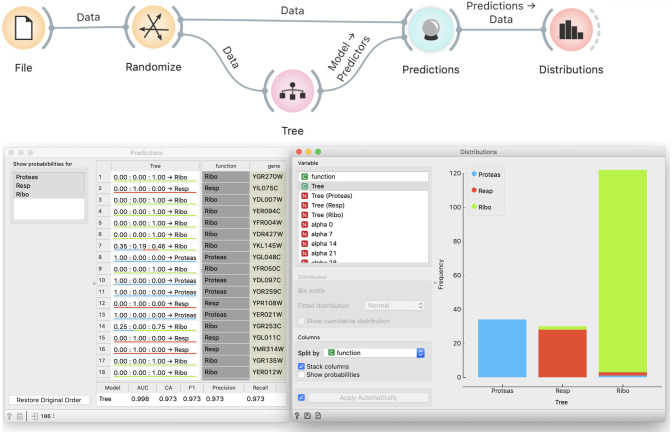
Modeling from permuted data. Permutation of class labels should prevent successful modeling, yet the Distribution widget and the scores at the bottom of the Prediction widget show that the tree almost perfectly fits the data.

To convincingly demonstrate that our procedure is wrong, we randomize the data through Randomize widget that we add after the File widget (Fig 3). Randomize widget permutes the class labels. We accompany this with a story about a lousy technician who mislabeled genes, thus ruining the data. At students’ request, we can also permute the values of independent variables, or, time allowing, we use the opportunity to discuss how this would change the data more fundamentally, destroying any structure in it, yet the effect we want to show does not require this.

Students agree that no algorithm should be able to learn from random data. Yet the Distributions widget shows that the tree’s predictions are still well-aligned with actual groups, and the classification accuracy remains high.

### Exploration of causes

**Fig 4:**
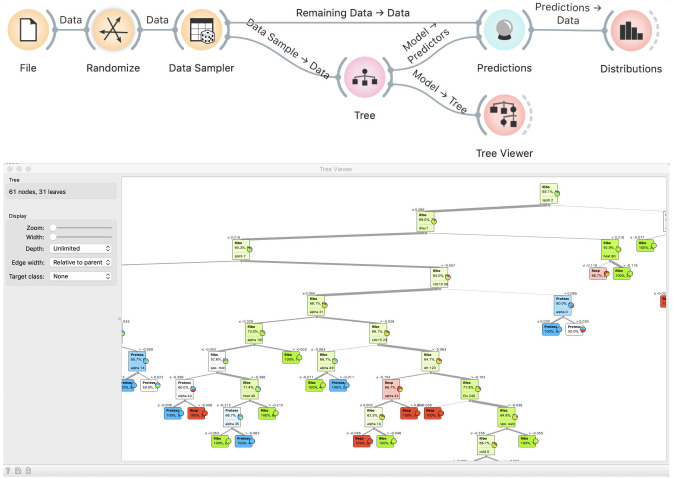
A tree induced from random data. Observing the tree reveals that it is too large for the given data set, and hence does not generalize well.

When asked why this happened, students will typically reply that it is because we are testing the model on training data. They will seldom offer the explanation of the exact mechanism. We instruct them to inspect the induced tree in a Tree Viewer (Fig 4). Observing the size of the tree, they realize that it effectively memorized the data. Typically, the discussion that follows is about the nature of modeling, namely, that modeling is about generalizing from the data. However, if the model remembers the data, it does not generalize and will not be useful on new, hitherto unseen data.

### Correct approach

**Fig 5:**
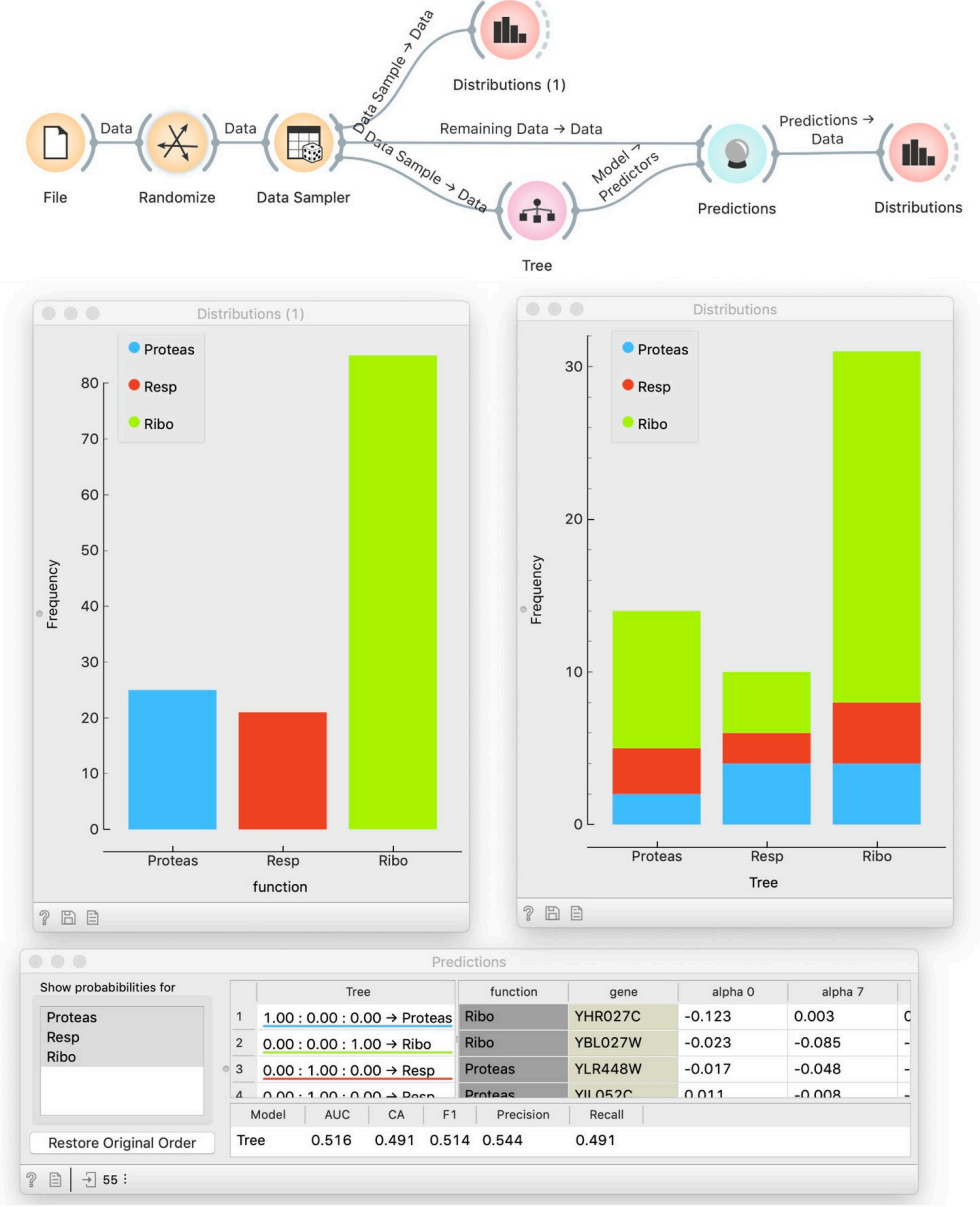
Testing a model on a separate data set. The Random Sample widget splits the data into two subsets, one for fitting and one for testing. Distribution of predictions by the model (right-hand histogram) roughly match the distribution of actual classes (left-hand side), but the actual class no longer matches the predicted.

This discussion leads directly to discovering of the proper way to test a model: using new data. We can do this by splitting the data into two subsets. This is done with the Data Sampler widget, where, for a better effect, we have to make sure that samples are stratified. We use sampled data for model fitting and out-of-sample data for testing (Fig 5). We now show two Distributions widgets: one that displays the class distribution in the training data and the other that shows predictions, as before. By comparing the two (which is why it is better to see stacked columns) we see that the distribution of predictions matches the distribution of target values in the training data–yet predictions have no relation with the actual class. Hence, using separate training and testing data can reveal overfitting.

Note how using a visual programming tool like Orange helps the educator: the workflow itself illustrated the procedure by graphically showing which data goes where.

**Fig 6:**
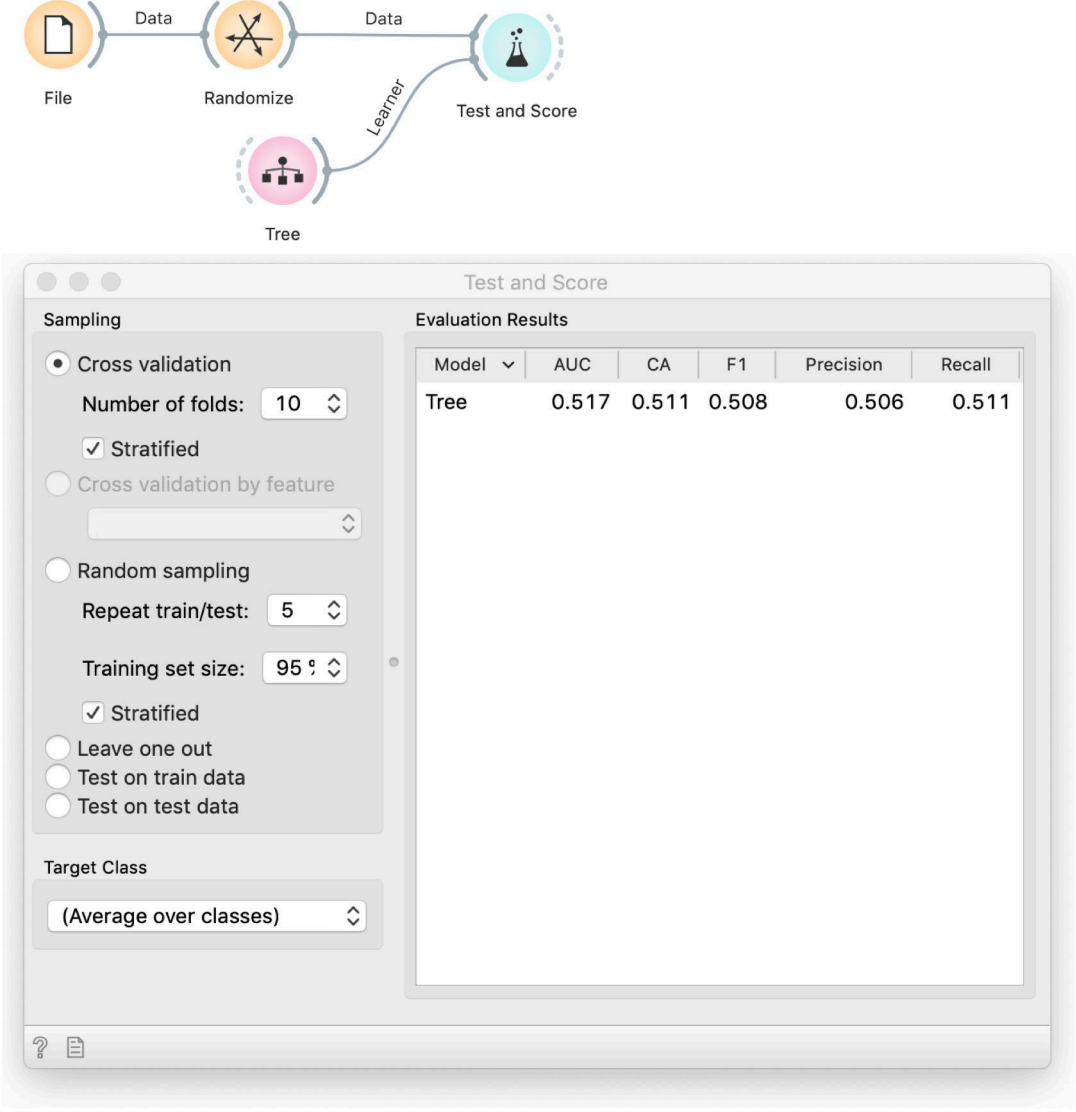
Testing with cross-validation. The Tree widget receives no data and does not output a tree but only an algorithm (ä recipe") for building one. The Randomize widget, which shuffles the data, is here only to demonstrate that cross validation discovers overfitting by showing a small accuracy. In practice, we would use the actual, non-randomized data.

Students may complain that this procedure depends on a single run of random sampling and thus on our luck. If they do not, we can raise the point ourselves to continue with an introduction of cross-validation. This leads to the workflow in Fig 6, which includes an element that has proven to be difficult, but conceptually essential to understand–the signal from Tree to Test and Score. Where does the Tree widget get the data? We need to explain that the model scoring widget, Test and Score, implements the entire cross-validation procedure. A *k*-fold cross-validation will fit *k* different models to *k* different (overlapping) data subsets. For this, it needs a learning algorithm, not a fitted model. While computer scientists see this as passing a function to a function, to non-computer scientists we explain that the Tree widget in the previous workflow outputs *a model*, while in this one, it has no data but can still output *a recipe for building a model*.

Stressing the difference between the trained model and the algorithm for model inference is important. It reminds students that cross-validation does not evaluate a model but the modeling procedure. The reported average score is not a score of any particular model.

### Take-home message

The mantra to always test models on separate test data has to be taken seriously. By violating it, we do not test model quality, but its ability to (over)fit the given data. This can lead to much larger performance over-estimates than most students realize.

### Limited perception of modeling

The former is a school example of overfitting that is unlikely to appear in respectable journals. In our next case, we show students a much more common problem. When taking care not to use the same data for modeling and testing, the modeling is often understood in a limited sense, for instance fitting the coefficients of logistic regression, and excludes any procedures like data preparation.

For this demonstration, we require a data set in which the number of variables largely exceeds the number of data instances. We will here use a dataset on breast cancer and docetaxel treatment [12] from Gene Expression Omnibus (dataset GDS360), which includes 9,485 genes (features) whose expression was observed in 24 tissue samples (data instances). This data set is indeed small; students need to be warned that smaller data sets of this kind often appear in medical research and that inference of reliable models would require more data or approaches that can additionally incorporate prior domain knowledge. Data instances are labeled with a binary class indicating weather the tumor was sensitive to treatment. Instead of (the already discredited) classifications trees, we use logistic regression, which, as a linear model, should be less prone to overfitting, making the example more convincing.

### Analytic procedure

**Fig 7:**
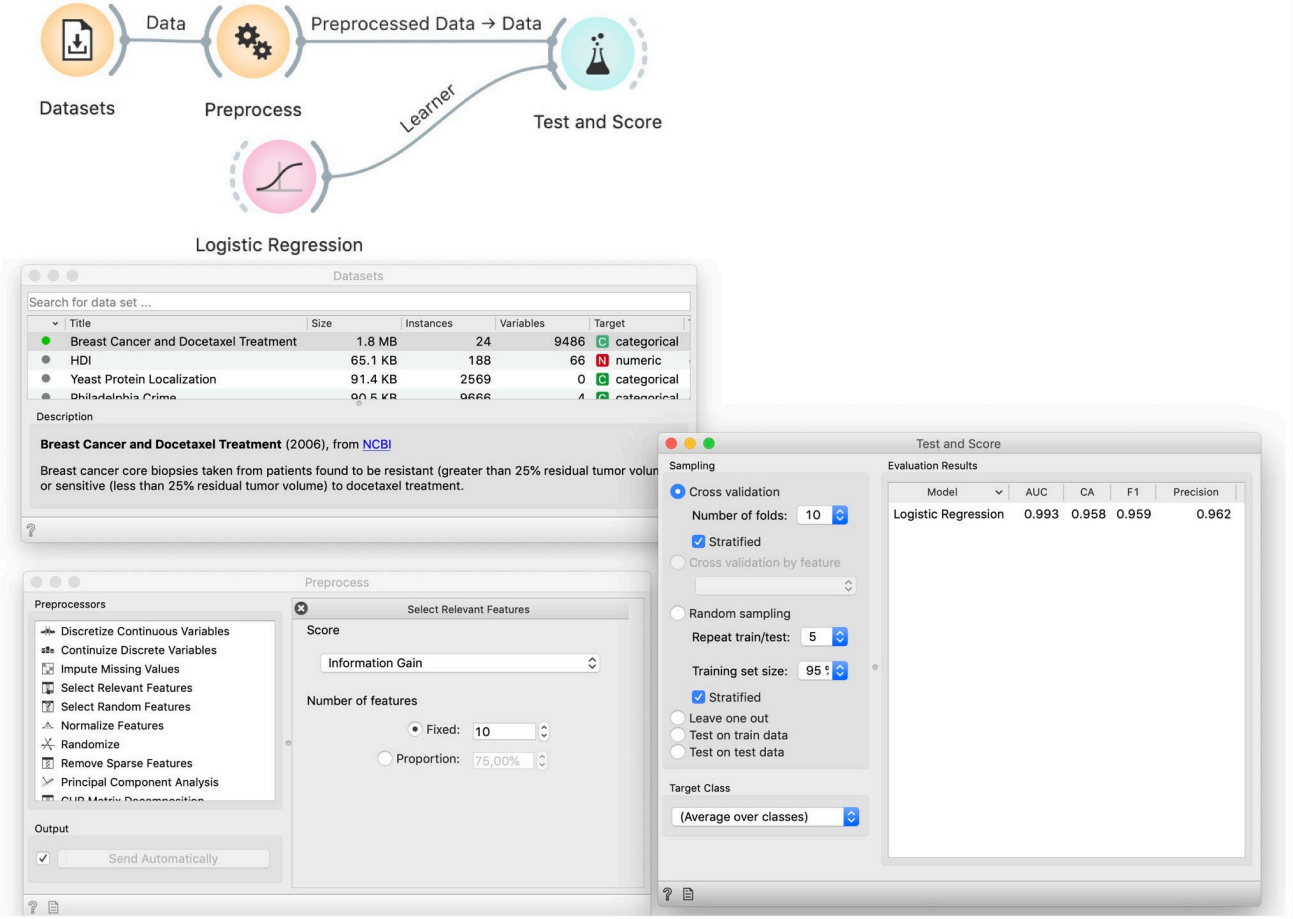
Improper way to select features. The Dataset widget loads the data from our curated repository of data sets. The Preprocess widget selects ten most informative features. This data is used to cross-validate logistic regression, which achieves a 96% classification accuracy.

We show the data and students easily agree that the model probably should not need to use all 9,485 variables. Selecting only a few should simplify the model, making it more robust, and speed-up its training. Based on experiences from the previous case, students also know that with fewer variables, models are less likely to overfit. We thus add Preprocess and use Select Relevant Features to choose ten variables with the highest information gain, that is, ten features that are most correlated with the binary class. We test the performance of logistic regression on this data in Test and Score (Fig 7).

To further (miss)lead the students down the sadly common path, we can invite them to discover the optimal number of variables. Depending upon random sampling, the best results on this data are usually achieved with around five variables.

### Demonstration of incorrectness

**Fig 8:**
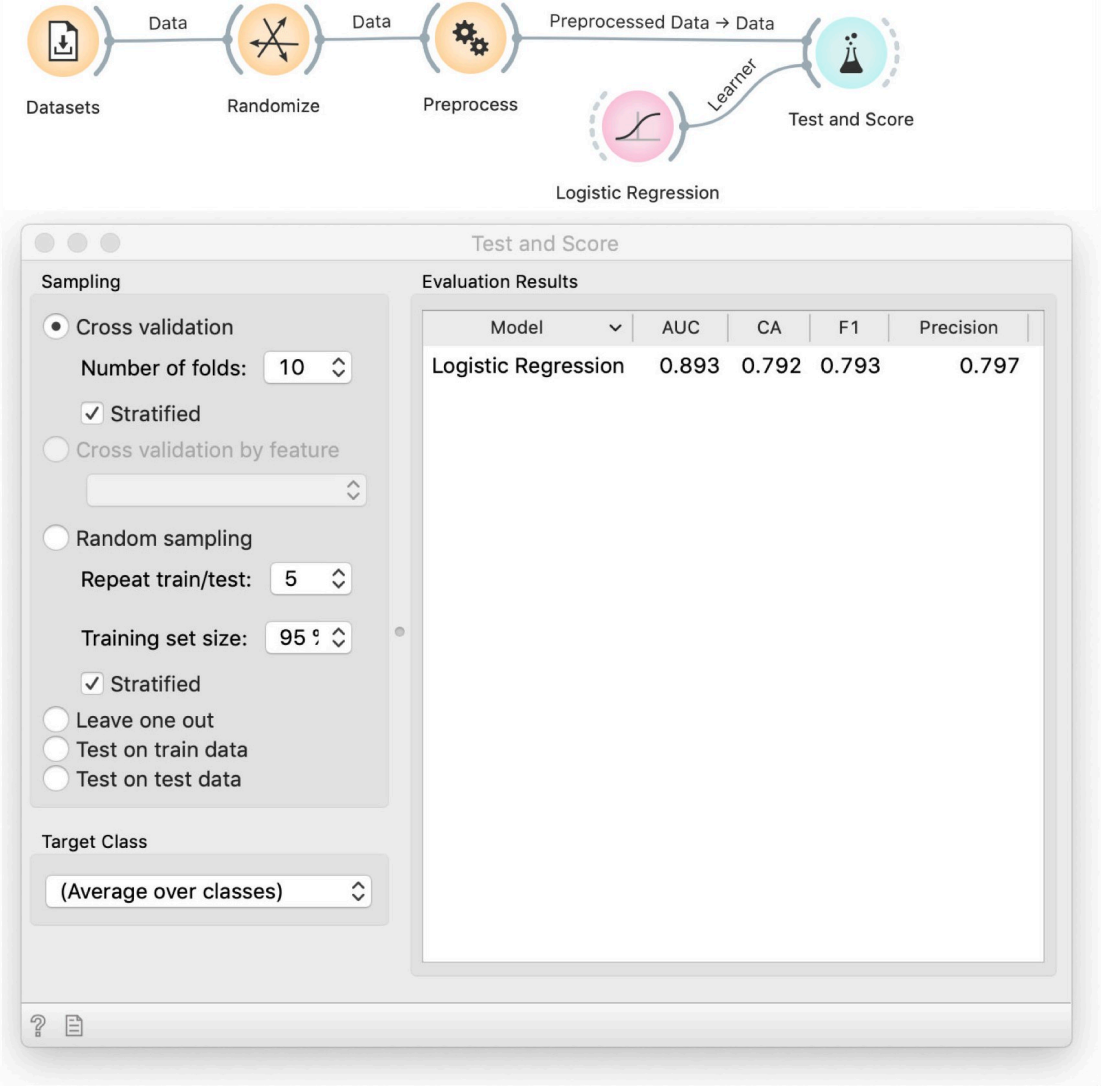
Selection of features on randomized data. Performance of logistic regression remains excellent even on randomized data: the classification accuracy is 80%, compared to the 60% majority class.

We use the same trick as before: we insert Randomize between File and Preprocess (Fig 8). This decreases the accuracy of logistic regression to around 80%, which is still better than the proportion of majority class (60%), thus the model performs quite well although the data is random.

### Exploration of causes

**Fig 9:**
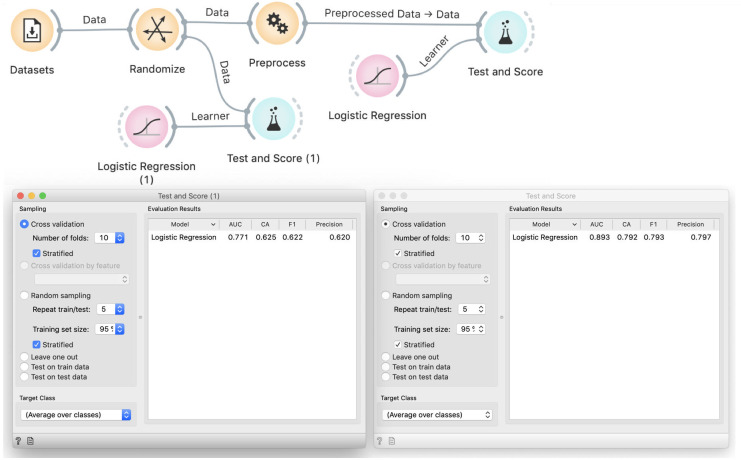
Comparison of models on all and on selected features from random data. Classification accuracy of logistic regression on all features is 62%, which is about the same as proportion of majority class. The model is thus no better the random guessing, as expected. This proves that feature selection is responsible for the overly optimistic result.

To hint at the problem, we ask the students to run cross-validation on randomized data with all features (Fig 9): the accuracy of logistic regression there is similar to the proportion of the majority class. With this, it is obvious that the source of the problem is the selection of informative variables. But–informative about what? The Preprocess widget selects variables correlated with the class: although the class has been randomized, we will encounter some randomly correlated variables just because there are so many to choose from. These correlations hold over the entire data set, so when the cross-validation splits the data set into training and testing samples, the correlation holds on both parts.

### Correct approach

**Fig 10:**
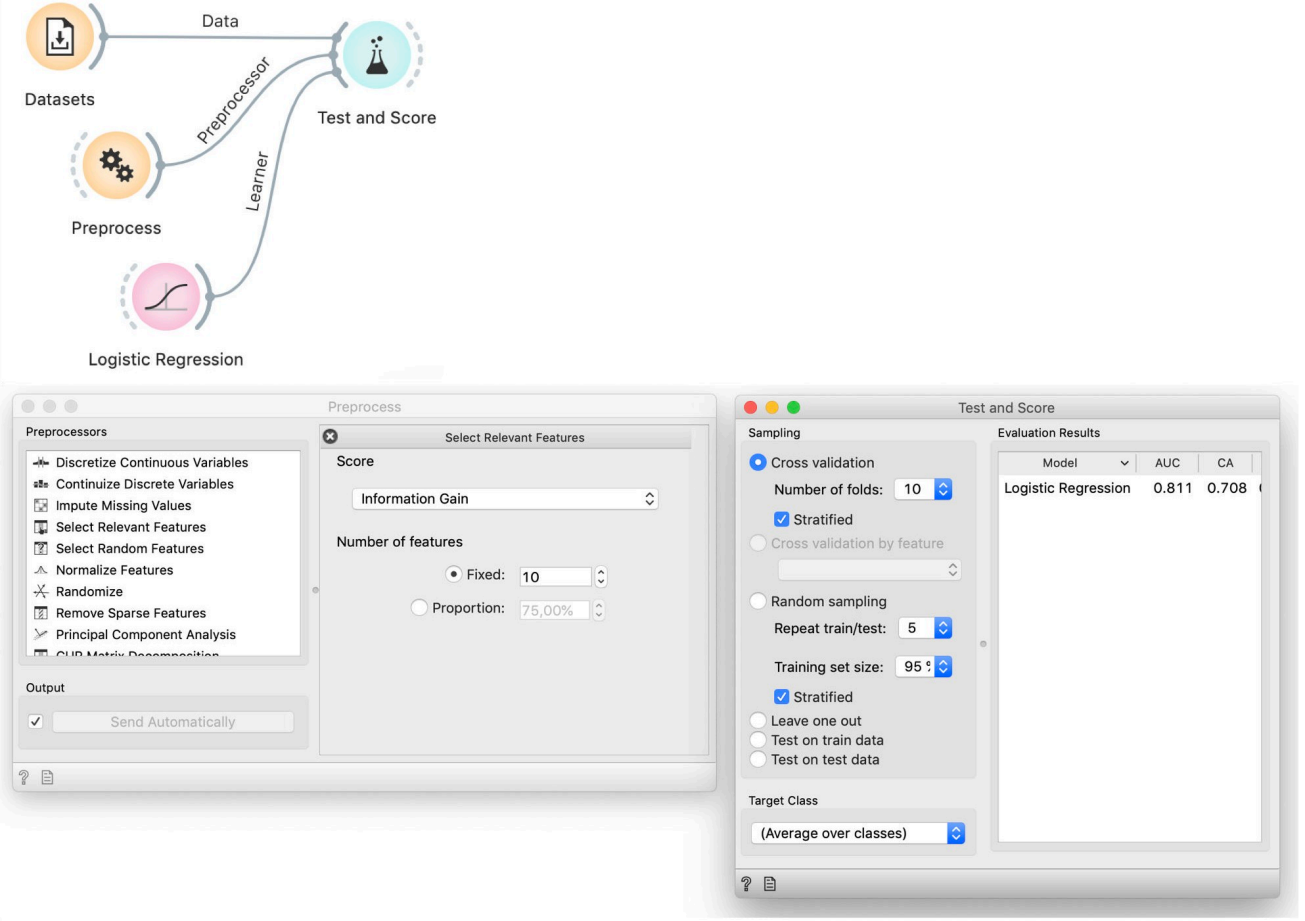
The proper workflow for cross-validation that includes data preprocessing. In this workflow preprocessing is not done prior to splitting the data. The preprocessing recipe, provided by the Preprocess widget, enters the cross-validation procedure and is applied to each training data subset separately, without being informed by the data that is used in testing.

The necessary fix is obvious: we should perform feature selection on the training data only. Given that k-fold cross validation uses *k* training subsets, each of them would have a different selection of features–which would only be "informative" for the training, but supposedly not for the testing data. To verify this idea in Orange, we provide the preprocessor as an input to the Cross Validation widget (Fig 10).

### Take-home message

Modeling is not limited to fitting the parameters of the model but includes all other procedures like data preprocessing, model selection, and hyper-parameter tuning, which we do before feeding the data into a learning algorithm. Data preprocessing may include feature selection, imputation of missing data based on statistics computed from the available data, binning, and normalization, which may all have substantial effects if we leak information from testing samples. Concerning model selection, the researcher may test a number of different learning algorithms using cross-validation and then report the best one. By doing so, (s)he fits the model selection to the data; on another random sample from the same data, this algorithm might be less successful, and another algorithm could be better. As before, we need another data set to assess the quality of the chosen model. Finally, simpler models may have only a few parameters, mostly found through model regularization. In contrast, for artificial neural networks, for instance, we have to construct its entire architecture, decide the number and types of layers, set the optimization-related parameters, and so forth. Those can also be overfitted–at least in principle–thus the chosen hyper-parameters need to be validated on a separate test set.

### Making nice visualizations

The above cases demonstrate how predictive models can find non-existing relations, and improper testing can mislead us to believe they are real. One could naively assume that visualizations cannot overfit because they do not model anything but merely show the data.

To challenge this assumption, we could use the same breast cancer data set as above, though the effect will be better with a somewhat larger data set. We will instead show a new useful widget: we will construct a data set using Random Data from Orange’s Educational add-on.

### Analytic procedure

**Fig 11:**
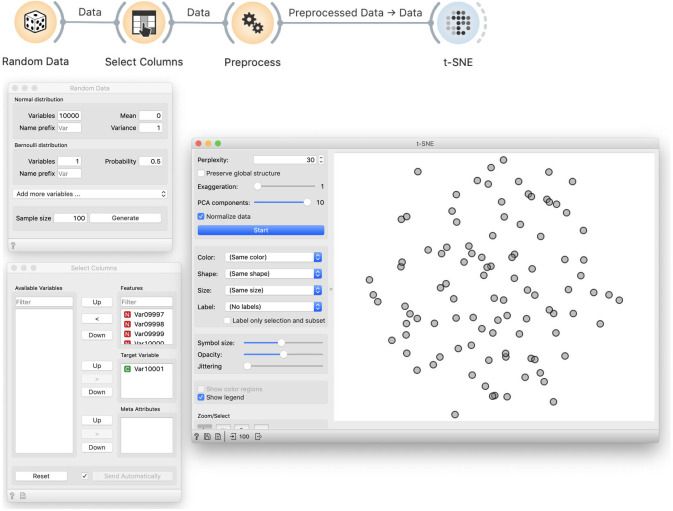
*t*-SNE visualisation of a random data. We generate 10,000 normally distributed random variables and one Bernoulli variable, which we designate as the target variable. In Preprocess, we choose ten variables that are most correlated with the target. This data is then used in the *t*-SNE visualisation. In this particular visualisation, the value of the target variable is not shown, and the dots in the t-SNE visualisation representing the data set items seem to placed randomly, as expected, and do not expose any clustering structure.

We create 10,000 normally distributed random variables and one Bernoulli variable with *p* = 0.5. The sample size will be 100. We then use Select Columns to designate the Bernoulli variable as the target, that is, the class variable. As before, we use Preprocess to select ten most informative variables. In other words, from a collection of 10,000 variables, we choose ten that are most correlated with the class variable. We then visualize this data using *t*-SNE. We get a cloud of randomly distributed points, as expected (Fig 11).

### Demonstration of incorrectness

**Fig 12:**
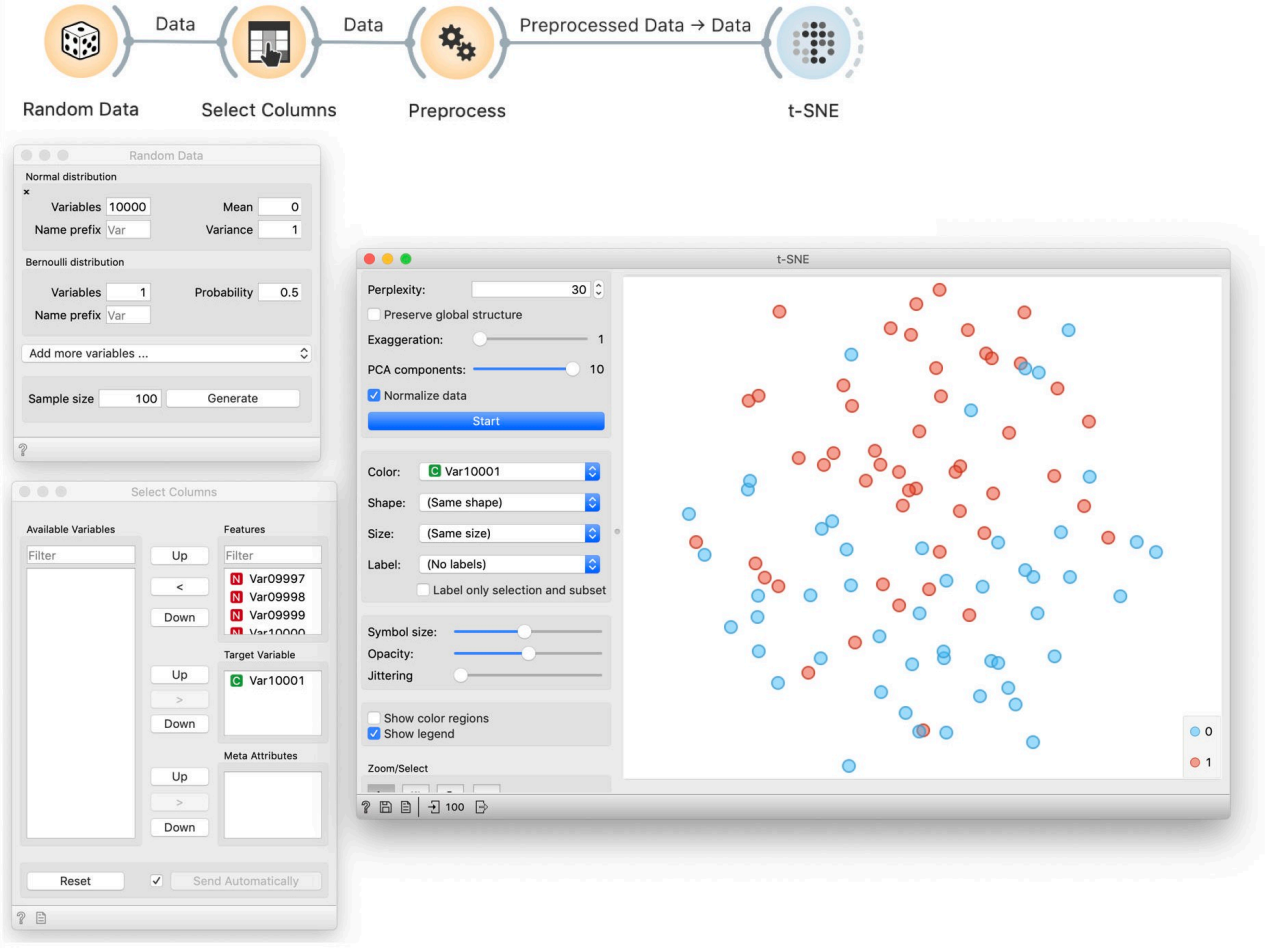
Colored *t*-SNE on random data. Data preprocessed by feature selection is visualized in a *t*-SNE plot that separates the data instances of different class, denoted by blue and red color. Density of blue data points is higher in the top part of the visualisation, and green points are denser at the lower half of the plot. This separation of instances of different class is seemingly surprising as the class value assignment is random, and is a by-product of preprocessing and choosing of ten features that are, albeit arbitrarily, most correlated with the class variable.

The problem with this approach is more subtle. Data visualization by *t*-SNE did not show any groups and hence worked correctly (Fig 11). To show that something is wrong, we color the points according to the assigned target value (Fig 12). We can see that *t*-SNE’s projection separates the two groups (though the separation is not perfect and depends upon random sample), which it should not be able to because the labels are random.

### Exploration of causes

The cause is similar to above: we again select features that are randomly related to the label. Yet the consequences differ: here we do not overfit a model, but we find order in chaos.

### Correct approach

**Fig 13:**
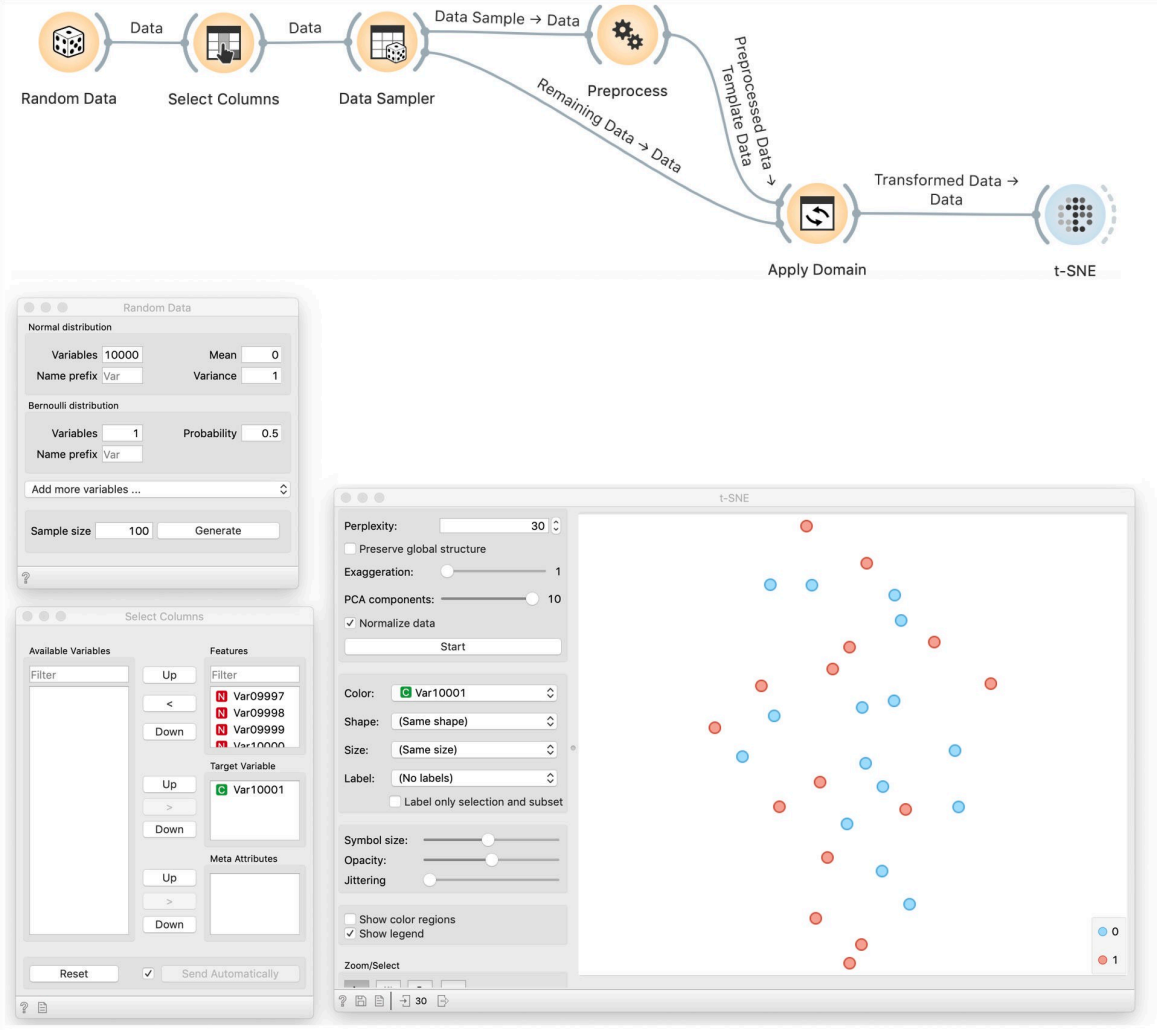
t-SNE visualization of a separate test data. This workflow uses a random sample to discover the most informative variables, that is, variables that are most correlated with the class variable. The Apply Domain then takes the out-of-sample data and applies the transformation from the Preprocess widget, and in this case, removes all except the ten variables chosen from the sample data. In this way, the procedure that selects the variables is not informed by the data that is shown in the visualization. This time, the data plot does not expose any class structure; red and blue data points are intermixed. Compare this outcome to the overfitted visualization from Fig 12.

The solution is similar to before: we split the data, use one part to select features, and then visualize the remaining data using only the selected features (Fig 13). Results on random data are then as bad as expected.

### Take-home message

While we cannot talk about overfitting visualizations for some particular performance score, their construction requires the same amount of caution as induction of models. Choosing a few features that are, albeit by chance, highly correlated with the class will always result in visualizations that expose class structure, even in the cases of random data.

## Discussion

Practical course on machine learning can be rewarding both to instructors and the audience. With the right tool, we can cover substantial ground and introduce new concepts in a hands-on workshop that can take only a few hours. Rewards for students come in the form of a plethora of new topics covered, and a-ha moments stemming from practical revelations on real data sets. Rewards for instructors come from students’ raised interest, and questions sparked from their engagement and interest in data exploration. Student engagement is rooted in the type of teaching that encourages them to do the data analysis while they learn, instead of just listening about it, as would be the case in a standard frontal, slides-based lecture.

We have introduced the hands-on lessons on overfitting that can be self-contained or a part of a longer machine learning course. For the latter, students would already know about data exploration, visualizations, and individual classifiers, like logistic regression, classification trees, and random forests. The required prerequisite of the course is also some understanding of the need for model evaluation. Prior knowledge about metrics to express model accuracy, such as precision, recall, and area under the ROC curve, would help the audience, but is not mandatory and could also be introduced during the proposed course. While we recommend that this course is a part of a series on machine learning, for a stand-alone workshop on overfitting, students could be encouraged to learn about Orange beforehand by watching a few selected introductory videos on Orange’s YouTube channel (http://youtube.com/orangedatamining).

The lessons would usually take two to three teaching hours, depending on the prior knowledge of the audience and the course placement. Standalone course could take three hours and further experiment with different data sets and classifiers.

**Fig 14:**
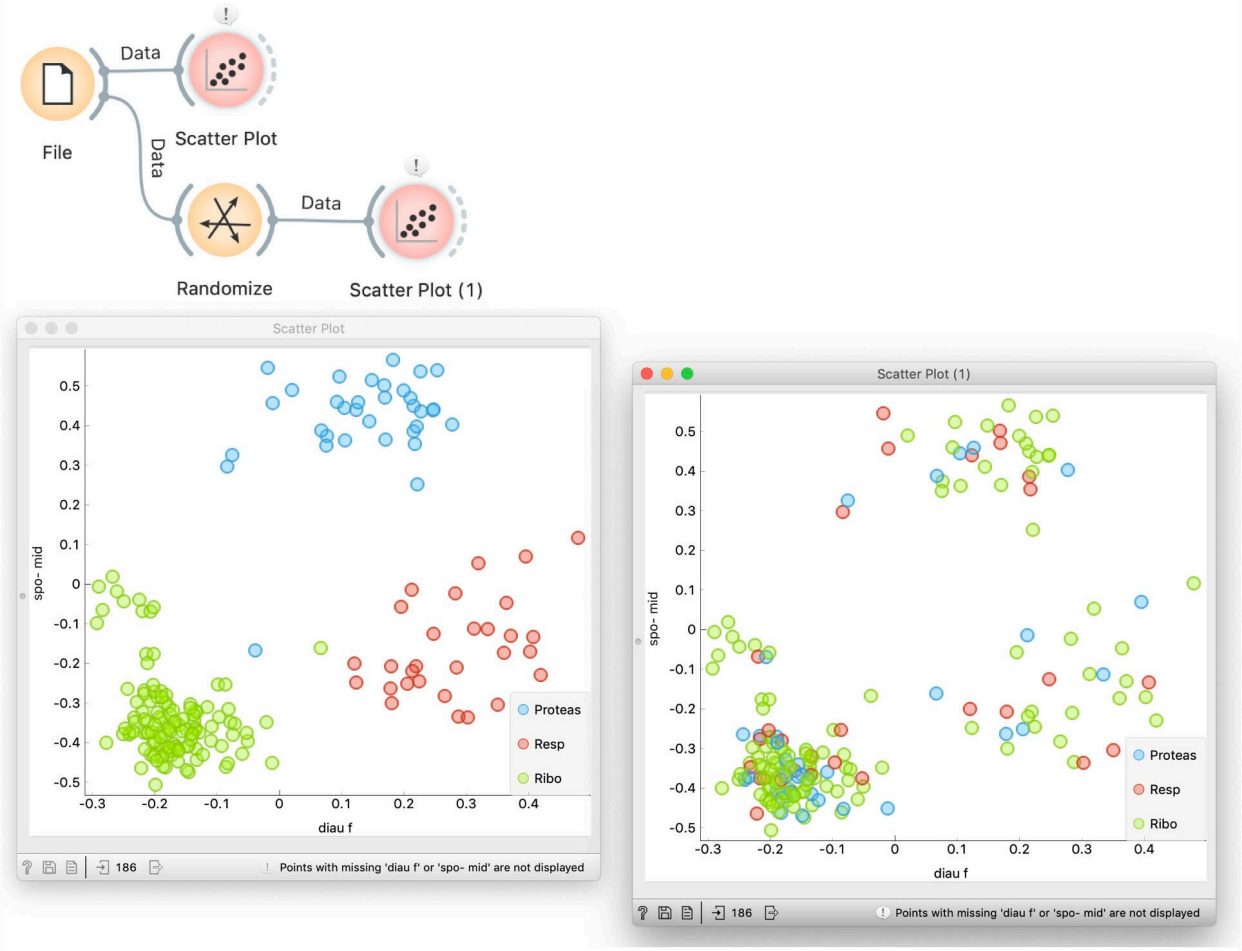
Exploring class-randomized data. Scatterplot in Orange can search for feature combinations that best split the classes. For the yeast expression data, diauxic shift (*diau f*) and sporulation at a five-hour timepoint (*spo-mid*) provide for the best combination. When the data is class-randomized, the class labels change, the pattern of class-separation is no longer there, but the data points keep their position. The effect of randomization is also visible by comparing the two Data Tables.

Especially for the audience who has just started exploring data science, the execution of the lectures could be slower and use additional explanations. For instance, students would often ask about the result of data randomization. How do we know that the data is indeed randomized? We can look at the data table. Still better, in the initial introduction of data set we can show that the yeast gene expression data has some structure and that for a specific combination of features, genes with different functions are well separated in the scatterplot (see Leban et al. [11] for automatic retrieval of such combinations). Shuffling of class labels will retain the position of points in the plot, but show the mixed classes (Fig 14).

Hands-on courses are different from ex-cathedra teaching. While the lead teacher is still on the podium, and uses, in our case, Orange to walk the students through the practical exercises, s(he) also frequently interacts with the students. Interactions can make sure that students go along, helping those who are stuck and solve any problems that arise. Students would often explore unplanned paths; these are often interesting and grant replicating on the projector to discuss with all participants. We recommend that for classes larger than five students, there is an additional instructor for assistance. The lecturer can often pause the narration to allow students to experiment on their own and synchronize the progress. While students may be given lecture notes in the form of workflows and explanations, just like the ones from this manuscript, their primary use is self-study after the workshop. The lecture does not involve any showing of slides. Everything is hands-on; Orange is the only tool shown on the projector. If an additional explanation is required, say, for classification trees, logistic regression, or similar, we recommend using the blackboard.

Courses oriented towards beginners must carefully balance between oversimplification and overwhelming. As we mentioned, our typical target audience is not students of computer science or math. Mathematically minded students may understand the topic easier if it is explained more formally. Computer scientists would benefit from being introduced to tools like Jupyter Notebook and the related Python machine learning stack or similar tools in other suitable languages. However, our target students, who do not possess those necessary skills, would find using programming tools more difficult than the concepts we intend to teach. The whole lecture may turn into a frustrating hunt for missing parentheses. In our past attempts at teaching data mining through programming in Python, we have been dissipating time to even such small details explaining that function names are case sensitive. The lessons we taught in this way were more about programming than about data mining. The environment we select to teach data science should serve as a helping tool instead of representing an obstacle to learning. Introduction to data science through textual programming either requires good coding skills or leads to a superficial understanding of the written code, which is in effect no deeper, but far less satisfying for students and teachers than using visual programming tools.

We use Orange in the lectures we propose here because of its strong focus on interactivity, simplicity of use, and educational value. In general, though, the tool’s choice depends on the teacher’s style, so we encourage the reader to explore other tools that support visual programming and workflows, such as Knime [13] or RapidMiner [14].

Using visual programming and avoiding mathematics exposes us to the danger of oversimplification. Students need to understand that they only attended an introductory course and that the expressiveness of visual programming languages, however powerful they may be, is very limited compared to coding in textual programming languages. More importantly, we need to clarify that the language of science is mathematics and not hand waving and nice pictures and that proper understanding can come only from studying the mathematical background. However, this is beyond what can be done in a few-hour lecture for the target audience we have assumed for the lectures presented here.

The students also need to be aware that they have been exposed to a limited set of techniques. This is usually not a problem because they notice that the used tools offer many options that we have not explored, such as sampling techniques other than cross-validation. It should also be evident that they were exposed only to school examples of carefully chosen data. In real-world applications, they would have to deal with many other problems like imbalanced data sets, missing data, and similar issues.

## Conclusion

Overfitting is one of the key concepts to be mastered in the training of machine learning. We here propose the content for a set of three lectures that introduce overfitting. The proposed lectures are hands-on, require students to work and explore the data while learning about machine learning concepts, and, to appeal to a broader audience, is carried out without the need of knowledge of any programming languages. This type of training is suitable for teaching the core, introductory classes to students with majors outside computer science, for instance, in molecular biology and healthcare.

Hands-on training is appealing for its immediate rewards. Students get instant feedback, gain understanding through exploring real data sets, and can relate to problems studied in class when later analyzing their own data. For depth, though, and where required, we have to combine such courses with more theoretical training. The training proposed here is not meant to replace standard courses in machine learning, but rather to complement them with up-front motivation and insight.
